# Genitourinary Diseases and Syphilis

**Published:** 1907-02

**Authors:** 


### BOOKS AND AUTHORS.

**Genitourinary Diseases and Syphilis.** By **Henry H. Morton,** M.D., Clinical Professor of Genitourinary Diseases in the Long Island College Hospital. Illustrated with 158 Half-tone and Photo-engravings and 7 Full-page colored plates. Second Edition, Revised and Enlarged. Royal Octavo, 500 Pages. Philadelphia: F. A. Davis Company. 1906. (Price, $4.00 net).

The worth of this book is best evidenced by the fact that a second edition has been found necessary. This is not only due to a general awakening as to the importance of genitourinary practice and a better and more rational understanding of its principles but also to the fact that the work approaches the ideal in make-up and in its presentation of the subject. There is every evidence that the author has gone carefully over the whole work and done much to improve it in the way of rewriting and amplification. He has kept pace with the advancements in the specialty and in the majority of instances gives the best and most up-to-date treatments for the ailments which beset the average genitourinary patient. Among the matters more fully gone into in this edition are surgical operations on the prostate and a clear expression of the pathologic anatomy and inflammatory affections of the organ. Much space is given to surgical diseases of the kidney, their diagnosis and treatment. It is a concise and helpful book, not very large as textbooks go, nowadays, but containing everything that the average worker in medicine need know; for those who wish to give more time than passing attention to genitourinary matters it is sufficient to lay a solid foundation for serious study and successful practice.

Naturally, Morton gives his personal views preference and there are times when his positive enthusiasm would have a tendency to mislead, as for instance, where he objects to the use of cocaine and penis ligation in circumcision. He prefers the clamp method and bases his objection to cocaine on the ground that the infiltration of tissues prevents cleanly operation and subsequent healing. In the writer's experience the cocaine method without the use of a clamp possesses none of the objections so seriously raised by Morton and the results have been so much better than with the clamp that the latter has been permanently discarded. It would appear better to leave the clamp method to the undisputed use of the rapid-fire general surgeon who rushes through a circumcision with the carless insouciance of the easygoing appendectomist. In meatotomy Morton advises that cocaine be injected into the frenum by hypodermic. That is hardly necessary if one aims to save the patient the least possible pain, and that should be one of the chief aims of the genitourinary surgeon. Meatotomies may be done without the slightest pain by inserting within the meatus a pledget of cotton caturated with 20% cocaine and allowing it to remain there three minutes before cutting. Still, these are minor matters and in no wise detract from the value of the book which, although it is smaller than many of the more pretentious works on the subject, is much more lucid than some of its bulky and heavy-lidded companions.

Especially excellent is the section devoted to the anatomy of the urethra; this is particularly intelligible and one does not flounder about in a maze of poor rewriting only to emerge from the chapter a-wonder at the ingenuity of human transposition.

Too much credit cannot be given Dr. Morton for the very clear manner in which he presents pseudo-gonococcic urethritis and the urethritis due to staphyloccocic and streptococcic infections. For this one section alone if for no other, the book would be well worth the study of the average general practitioner who makes his diagnosis and outlines a treatment on the mere visual inspection of a drop of pus at the meatus. There are some new ideas in the treatment of chancroid and its complications, but one must question the author's general condemnation of the dissecting out of enlarged ingenial nodes prior to suppuration. He prefers to allow the node to go on to suppuration, and then make a very small incision and inject the cavity with iodoform glycerine, thus curing the bubo in "five or six days." This is an excellent method—in some cases; but there is nothing logical or practical in the sweeping condemnation of the cleaning oft of the groin. Morton says a condition simulating elephantiasis of the penis and scrotum follows this procedure as a result of interference with lymphatic circulation. In something over a hundred dissections recently done under local and general anesthesia, there has been not the slightest sign of such a sequel; on the contrary the results have been uniformly successful and healing has been rapid and uncomplicated.

In spite of thes minor details of personal preference and individual practice in procedure the book stands without a peer among the smaller works and in some respects it surpasses many of the larger volumes. The appendix is a mine of information, detailing as it does laboratory technic covering the examination of secretions of every character and the preparation and staining of slides for tubercle bacilli, spermatozoa, and the spirocheta pallida.

The illustrations are excellent, many of them being from the authors' cases. There are 158 half tones and seven full page plates in color which are most artistic. Dr. Morton deserves hearty congratulations on the manner in which he has put together the book and for the very interesting manner in which he has handled a most difficult specialty in medicine.

